# Neurological events related to influenza A (H1N1) pdm09

**DOI:** 10.1111/irv.12241

**Published:** 2014-02-13

**Authors:** Graciela Cárdenas, José Luis Soto-Hernández, Alexandra Díaz-Alba, Yair Ugalde, Jorge Mérida-Puga, Marcos Rosetti, Edda Sciutto

**Affiliations:** a Department of Neuroinfectology, Instituto Nacional de Neurología y Neurocirugía Manuel Velasco SuárezMexico City, Mexico; b Instituto de Investigaciones Biomédicas, Universidad Nacional Autónoma de MéxicoMexico City, Mexico

**Keywords:** AH1N1 pandemic, influenza, neurological events

## Abstract

**Objectives:**

To review neurological complications after the influenza A (H1N1) pdm09, highlighting the clinical differences between patients with post-vaccine or viral infection.

**Design:**

A search on Medline, Ovid, EMBASE, and PubMed databases using the keywords “neurological complications of Influenza AH1N1” or “post-vaccine Influenza AH1N1.”

**Setting:**

Only papers written in English, Spanish, German, French, Portuguese, and Italian published from March 2009 to December 2012 were included.

**Sample:**

We included 104 articles presenting a total of 1636 patient cases. In addition, two cases of influenza vaccine-related neurological events from our neurological care center, arising during the period of study, were also included.

**Main outcome measures:**

Demographic data and clinical diagnosis of neurological complications and outcomes: death, neurological sequelae or recovery after influenza A (H1N1) pdm09 vaccine or infection.

**Results:**

The retrieved cases were divided into two groups: the post-vaccination group, with 287 patients, and the viral infection group, with 1349 patients. Most patients in the first group were adults. The main neurological complications were Guillain-Barre syndrome (GBS) or polyneuropathy (125), and seizures (23). All patients survived. Pediatric patients were predominant in the viral infection group. In this group, 60 patients (4.7%) died and 52 (30.1%) developed permanent sequelae. A wide spectrum of neurological complications was observed.

**Conclusions:**

Fatal cases and severe, permanent, neurological sequelae were observed in the infection group only. Clinical outcome was more favorable in the post-vaccination group. In this context, the relevance of an accurate neurological evaluation is demonstrated for all suspicious cases, as well as the need of an appropriate long-term clinical and imaging follow-up of infection and post-vaccination events related to influenza A (H1N1) pdm09, to clearly estimate the magnitude of neurological complications leading to permanent disability.

## Introduction

In 2009, a new strain of swine-originated influenza A (H1N1) caused the first pandemic of the 21st century.^1^ Clinical manifestations of influenza A (H1N1) pdm09 infection ranged from mild symptoms to severe illness and death. Most patients with severe or fatal disease were reported to have underlying medical conditions, including chronic lung disease, diabetes, cardiovascular disease, neurological disease, and pregnancy;^2^–^4^ nevertheless, some individuals developed neurological complications without having underlying comorbidity. In fact, neurological complications associated with either vaccination or infection events are well described in influenza.^5^–^7^

Here, we present a literature review of influenza-related neurological complications related to influenza A (H1N1) pdm09, highlighting the different clinical outcomes between those patients who developed neurological complications after vaccination and viral infection, respectively. Additionally, two vaccine-related neurological complications observed in our own neurological care center in Mexico are reported.

## Methods

### Literature review from March 2009 to December 2012

We conducted systematic searches in Ovid, Medline, EMBASE, and PubMed databases using the keywords “neurological complications of Influenza AH1N1” or “post-vaccine Influenza AH1N1” to identify published papers on this topic. Two clinical neurologists (C.G. and D.-A.A.) performed a full-text review of the papers and extracted all relevant data. Inclusion criteria for this review included studies reporting basic clinical data on influenza-related neurologically defined events, laboratory data confirming influenza A (H1N1) pdm09 infection and at least age-group information (adults versus pediatrics), because in most cases, a precise age was missing. In those cases reporting the age, pediatric patients were considered as <16 years old, both for infection and post-vaccine events. Only papers written in English, Spanish, German, French, Portuguese, and Italian were included. Those papers written in other languages or poorly written because of lacking relevant clinical and epidemiological data were excluded. Additionally, two adult patients with acute disseminated encephalomyelitis (ADEM) post-influenza A H1N1 vaccination studied at a Mexican neurological care center are reported.

### Statistical analysis

Data analysis was performed using the Statistical Package for the Social Sciences (IBM SPSS statistics 20 Inc., Somers, NY, USA). Qualitative variables were expressed in percentages and compared using chi-square test with Yates correction. Differences between continuous variables were evaluated using a Student's *t*-test. A *P* < 0·05 was considered as significant. Charts were created using the R platform. http://www.r-project.org/

## Results

A total of 115 articles were initially retrieved, but eleven of them were eliminated according to our exclusion criteria. Finally, considering our inclusion criteria, only 104 articles (1636 patients) were included and divided into two groups, according to the origin of neurological complications: influenza vaccine-related (287 patients) or viral infection-related (1349 patients) (Tables S1 and S2).^5^–^108^ The main characteristics of patients are summarized in Table 1.

**Table 1:** Neurological complications due to Influenza A (H1N1) pdm09 post-vaccination and infection

	Post-vaccine (%)	Infection (%)	*P*
Groups			
Pediatric	37 (2.9)	1256(97.1)	0.0001*
Adult	250 (72.9)	93 (27.1)	
Time of clinical symptoms (days)	8.73 ± 7.8	3.17 ± 2.8	0.06**
Clinical manifestations			
GBS or cranial neuropathy	125 (64.1)	70 (35.9)	0.0001*
Seizures and status epilepticus	23 (5.9)	369 (94.1)	0.0001*
ADEM	20 (71.4)	8 (28.6)	0.0001*
Encephalitis-Encephalopathy	41 (8.0)	471 (92)	0.0001*
Stroke	7 (36.8)	12 (63.2)	0.1*
Myelitis	3 (42.9)	4 (57.1)	0.1*
Others	68 (12.7)	469 (87.3)	0.0001*
Clinical outcome			
Improvement	287 (19.1)	1218 (80.9)	0.0001*
Death	0	60 (100)	
Total of patients registering clinical outcome	287	1349	

GBS, Guillain-Barré syndrome; ADEM, acute disseminated encephalomyelitis. Statistically significant differences are in bold.

* Chi-square test with Yates correction,

** Student's *t*-test.

### Neurological complications related to post-vaccine events

Post-vaccine-related neurological complications were observed in 287 patients. From these, 54 were male and 14 female, and there was no information on gender for the remaining patients (219). Mean age was 30·16 ± 25 years. Neurological complications consisted mainly of Guillain-Barré syndrome (GBS) or, polyneuropathy (125), seizures (23), acute disseminated encephalomyelitis (ADEM) (20), encephalopathy-encephalitis (41), stroke (7), transverse myelitis (3), and others (68). All patients survived and permanent sequelae were reported in 2 (0·69%). CSF-cytochemical characteristics showed 23·55 ± 31·8 cell/μl, 88 ± 28·8 mg/dl of glucose, and 45·97 ± 23·7 mg/dl of protein. While few reports and series provided a neuroimaging pattern, demyelinating lesions were predominant in this group.

### Neurological complications related to infection events

Infection-related neurological complications were observed in 1349 patients. Mean age was 12·75 ± 14·6 years. Two hundred and ninety-four were male and 196 female; there was no gender information on the 855 remaining patients. One-thousand two hundred and fifty-six patients (93%) were pediatric and 93 were adults. Forty-nine patients showed previous co-morbidity, mostly previous neurologic disease such as anoxo-ischemic cerebral sequelae, febrile seizures, and myasthenia. In this group, 60 patients (4·7%) died and 52 (30·1%) developed permanent sequelae. CSF-cytochemical characteristics were 67·99 ± 253·5-cells/μl, 63·62 ± 27·5 mg/dl of glucose, and 118·75 ± 381·3 mg/dl of proteins. In 49 patients, previous co-morbidity factors such as obesity, asthma, or other chronic diseases were present. A wide spectrum of neurological complications was observed: encephalopathy-encephalitis (464), seizures or epileptic status (369), stroke (12), ADEM (8), transverse myelitis (4), and others (469).

### Comparison between vaccine-related and infection-related events

Some important clinical features registered in both groups were pregnancy, obesity, asthma, and other chronic diseases in 170 patients. Adults were more frequently affected in the post-vaccine group, 250 (72·9%), while children predominated in the infection group, 1256 (97·1%), chi-square value = 914, *P =* 0·0001. Clinical outcome was less severe in the post-vaccine group than in the infectious group, where more cases of permanent sequels and deadly events were recorded. GBS was prevalent in the post-vaccine group (64·1% versus 35·9%) chi-square value = 328, *P* = 0·0001), whereas the encephalopathy-encephalitis spectrum predominated in the infection group (92% versus 8%) chi-square value = 45·8, *P* = 0·0001). It is important to note that deaths were recorded in the viral infection group only, most of them in pediatric age band, 50 of 60 (83·3%). Neuroimaging analysis revealed two main patterns: demyelinating and vascular. The main differences between both groups are summarized in Table 1.

### Illustrative cases

#### Patient 1

**Figure 1:**
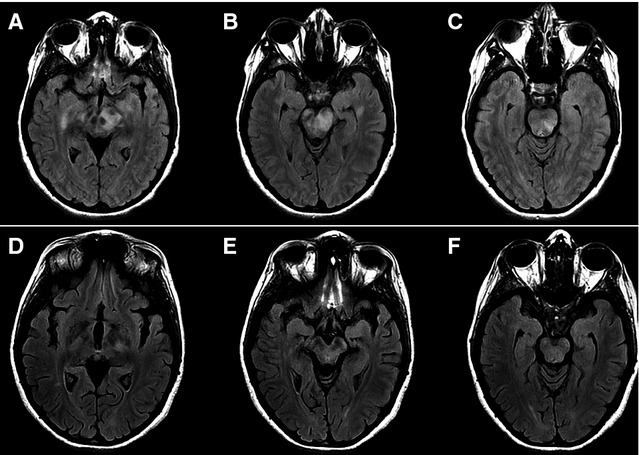
(A–C) MRI on axial Fast-FLAIR sequence shows demyelinating areas involving gyri rectus, mesencephalon, and pons. (D–F). Control MRI performed 15 months after initial symptoms shows improvement in demyelinating lesions and secondary cortical atrophy.

A 27-year-old woman with a history of atopic dermatitis received monovalent inactivated influenza A (H1N1) vaccination on December 2009; 4 weeks later, she presented cephalgia, generalized weakness, progressive left-sided hemiparesis, and hypersomnia. She was evaluated at another institution. CT scan revealed brain edema. CSF-cytochemical analysis showed 81 mg/dl of glucose and 31 mg/dl of proteins without cells. Bacterial and tuberculosis cultures were negative. Serological test against Coxsackie virus, CMV, and cysticerci, as well as CSF-PCR for herpesvirus, were negative. Acute phase reactants were normal. She was treated with acyclovir and corticosteroids for 21 days, with some clinical improvement. Four weeks later, she showed abrupt changes in social behavior and dromomania (an uncontrollable psychological urge to wander). Clinical deterioration progressed with disarthria, paresthesias, and consciousness impairment. Upon admission to our Institution, neurological examination revealed stupor, left-central facial paralysis, hypertonic reflexes, grasp reflexes, persistent postures, generalized rigidity, and a left Babinski sign, making up a catatonic syndrome. MRI scan showed demyelinating lesions involving bilateral basal ganglia and brainstem (Fig.1A–C). A new evaluation with cultures and serological tests was not contributory. The patient received corticosteroids, olanzapine, and lorazepam, with slow improvement. She was discharged 3 weeks later. As an outpatient, complex neuropsychiatric symptoms appeared with hyperorality, hypermetamorphosis (an irresistible impulse to notice and react to everything within sight), and hypersexuality, also known as Klüver-Bucy syndrome. A control MRI scan performed 15 months later showed radiological improvement (Fig.1D–F). During a 27-month follow-up, the patient required multiple hospitalizations at a psychiatric ward for depression and impulsivity with suicidal ideation. She is still under antipsychotic treatment and requires continuous supervision by relatives.

#### Patient 2

**Figure 2:**
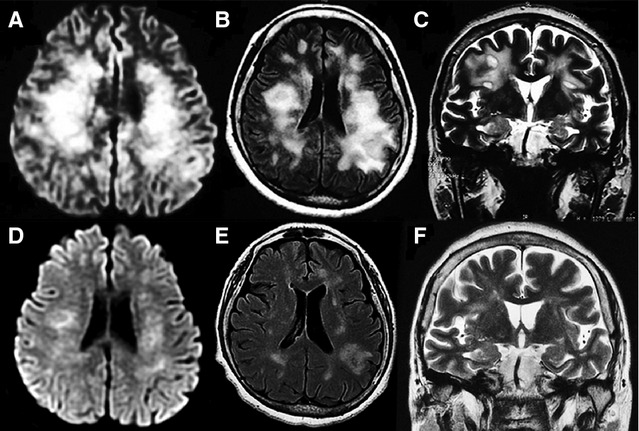
(A–C) MRI on diffusion, Fast-FLAIR, and T2-weighted sequences show extensive demyelinating areas involving subcortical white matter (bilateral centrum semi oval). (D–F) Control MRI performed 4 months after initial symptoms shows ostensible improvement of lesions and secondary subcortical atrophy.

A 64-year-old woman was referred with a 2-month history of treated arterial hypertension and vitiligo, diagnosed 6 months before. She received trivalent inactivated A (H1N1) influenza vaccination on November 2011. One month later, she developed irritability, semantic paraphasias, and memory impairment. Upon neurological examination she was alert, oriented with euthymic mood, low fluent spontaneous speech, and increased latency of verbal responses. She was unable to keep or focus attention; constructional praxis and calculation-abstraction were seriously affected. Clinical findings were consistent with a frontal lobe syndrome. Electroencephalogram showed mild generalized dysfunction. CSF-cytochemical analysis rendered 82 mg/dl of glucose and 26 mg/dl of proteins without cells. PCR-viral panel for herpesvirus family was negative, as well as VDRL, cryptococcal antigen, bacterial, fungal, and mycobacterial cultures. Other evaluations, including HIV serology, IgM serology for EBV, CMV, antinuclear antibodies, and antineuronal antibodies, were all negative. A first MRI scan taken 1 month after the initial symptoms showed diffuse cortico-subcortical white matter involvement and edema (Fig.2A–C). Patient was treated with intravenous methylprednisolone for 3 days, followed by oral tapering corticosteroids. On a control MRI scan, 4 months after the symptoms onset (Fig.2D–E), a significant reduction in white matter abnormalities was seen. Neurological improvement has been progressive, and at 6 months of follow-up, the patient still receives rehabilitation and psychotherapy).

## Discussion

Although influenza virus A (H1N1) pdm09 has become a public health threat of global concern,^109^,^110^ there are no accurate data regarding the worldwide frequency of neurological complications related to this disease. Here, we reviewed the reported neurological complications from this disease from pandemic onset until December 2012; we found more reports of neurological complications due to the infection itself than to vaccination. The Vaccine Adverse Event Reporting System estimated that from October 2009 through January 2010, 82·4 million doses of 2009 H1N1 vaccine were administered; death, GBS, and anaphylaxis reports after 2009-H1N1 vaccination were rare (<2 per million doses administered, each).^111^

On the other hand, WHO aimed to provide access to A (H1N1) pdm09 vaccine for all countries as soon as the vaccine was available and approved. Each recipient country established its vaccination programs, with some degree of variation depending on national priorities. Some chose to vaccinate only specific priority groups, including at least some children and younger adults (including pregnant women), and healthcare workers.^112^–^118^ According to our review, fatal cases and permanent neurological sequelae were reported in about 11% of patients with influenza-related neurological complications. With respect to vaccination, aging appears to be related to a higher risk of having neurological sequelae, as in our two reported cases. The clinical outcome was generally more favorable in post-vaccine neurological complications. In fact, no fatalities occurred in this group, but permanent sequelae were observed in 2/287 patients, our two ADEM post-vaccine cases, patients were female and developed severe and complex neuropsychiatric sequelae.

Most patients with neurological complications related to influenza A H1N1 infection developed necrotizing or non-necrotizing encephalopathy; an antecedent of chronic diseases was found in some of these patients.^8^,^9^,^14^,^23^,^31^,^37^,^47^,^48^,^50^,^62^,^64^ A number of individual factors such as the patient's age may be involved; children and elderly are the groups with the highest risk of having neurological complications, but also are those suffering underlying immune disorders, chronic diseases, or obesity.^119^,^120^ In our review, most neurological complications occurred in the population younger than 16 years (1297 versus 342 cases) Graph.1. This result, along with the pivotal role of the young in spreading the infection, highlights the relevance of enforcing vaccination in this population group, where severe post-vaccination complications are infrequent. There are insufficient details in the published reports about neurological complications related to influenza A (H1N1) infection or vaccination to reveal other individual and circumstantial factors that may participate in the susceptibility or development of such complications, and their optimal management is unknown.

Large population studies of risk factors for influenza have been conducted in both seasonal and influenza A (H1N1) pdm09.^119^,^120^ The highest hospitalization rates were found in children; in contrast, higher mortality rates were found in persons over 64 years old. Even though aged population may have a lower risk of infection, they showed a higher risk of death when infected.^121^

With respect to pathogenesis, while influenza viruses do not seem to show a direct tropism to the nervous system, virus detection within both retina and the olfactory bulb has been described in animal models.^121^,^122^ Influenza virus has been also detected by isolation or nested RT-PCR in human cerebrospinal fluid^59^,^74^,^123^,^124^ on brain tissue in neuropil, ependyma, Purkinje cells, and other neurons.^125^ Although in most influenza cases, no virus has been detected in CSF, the reported information stresses the relevance of searching for the virus in the central nervous system in different infection stages, particularly when neurological symptoms are present.

On the other hand, other pathogenesis factors may be linked with neurological disorders related to influenza vaccination. As it is reported, the vaccine itself may promote an exacerbated peripheral inflammatory response,^126^ the extension of which may be modulated by individual biological factors, that is, age, sex, and genetic background. Furthermore, an increased systemic or peripheral inflammatory response may promote neuroinflammation, which may underlie the neurological symptoms observed in the two cases reported herein, and in those published elsewhere.^127^

In this regard, the severity of brain dysfunction even in cases with non-clinical neurological findings may be correlated with high levels of pro-inflammatory cytokines in blood and CSF (cytokine storm).^128^ However, in some cases of CNS involvement, no cytokine storm or tissue inflammatory infiltrate has been found.^63^,^129^ It is also possible that both the viral infection and the vaccination promote blood–brain barrier (BBB) dysfunction,^130^ producing neuroinflammation and neurological disorders. With respect to neurological diseases related to the infection, it is important to consider the higher prevalence of encephalopathy or encephalitis in the pediatric population. The size of the influenza viral particle may prevent it from crossing the barrier in adults, but an immature BBB may be prone to virus invasion.^103^

Even though our analyses show clear limitations due to the incomplete information in most of the case reports retrieved from medical literature, and also to their descriptive nature, the information reviewed in this article highlights the relevance of an accurate neurological evaluation in all suspicious cases and of an appropriate long-term clinical and imaging follow-up of infection and post-vaccination events related to influenza A (H1N1)pdm09, to clearly estimate the magnitude of neurological complications that could lead to permanent disability.

## Supporting Information

**Graphic S1.** References included in the review Neurological events related to Influenza A (H1N1) pdm09.

**Table S1.** Description of the different neurological symptoms related to influenza vaccination. **Table S2.** Description of the different neurological symptoms related to influenza infection.
