# Supplementary figures and images for: Neurological events related to influenza A (H1N1) pdm09
Source: Influenza Other Respir Viruses. 2014 Feb 13;8(3):339–46. doi: 10.1111/irv.12241 (PMC4181482; doi:10.1111/irv.12241)

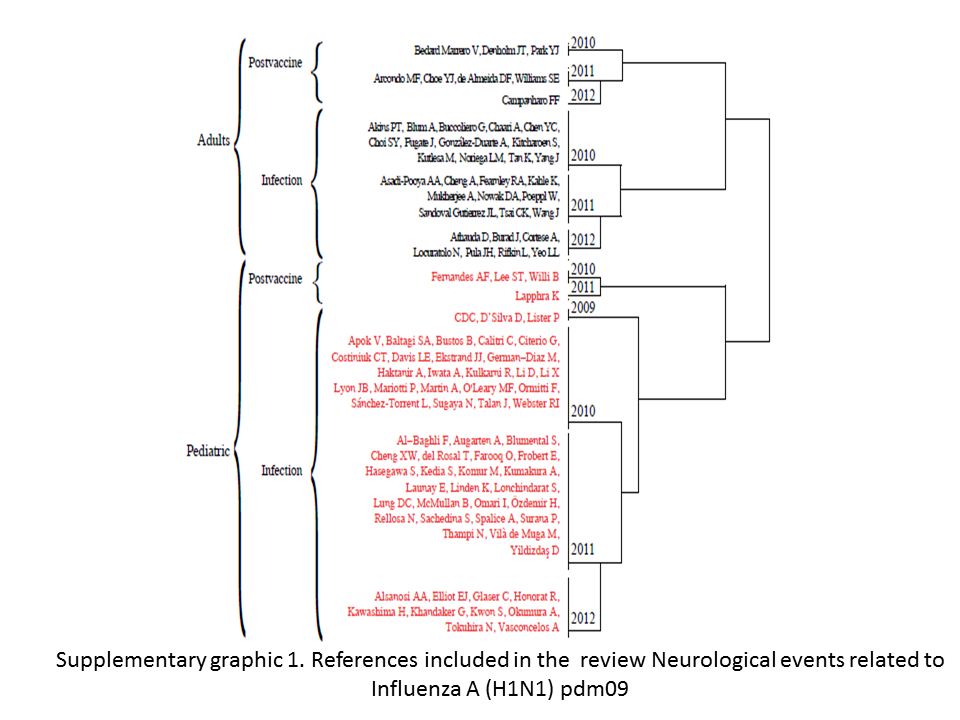

Supplement: Supplementary file 1 — Graphic S1. References included in the review Neurological events related to Influenza A (H1N1) pdm09. [file irv0008-0339-SD1.tif]
