# Supplementary material for: Neurological events related to influenza A (H1N1) pdm09
Source: Influenza Other Respir Viruses. 2014 Feb 13;8(3):339–46. doi: 10.1111/irv.12241 (PMC4181482; doi:10.1111/irv.12241)
Supplement: Supplementary file 2 [file irv0008-0339-SD2.doc]

**Supplementary Table 1. Description of the different neurological symptoms related to influenza vaccination**

|  |  | **Group of patients** | | **Post-vaccine neurological events** | | | | | | | **Clinical outcome** | |
| --- | --- | --- | --- | --- | --- | --- | --- | --- | --- | --- | --- | --- |
| **Reference/Country** | **Number of patients** | **Pediatrics** | **Adults** | **#CN-GBS** | **#S-ES** | **#ADEM** | **#Encefalitis-ANE** | **#Stroke** | **#Myelitis** | **#Others**** | **#Survived** | **Died** |
| Bedard Marreno V, 2010/Puerto Rico | 2 | 0 | 2 | 2 | 0 | 0 | 0 | 0 | 0 | 0 | 2 | 0 |
| Denholm JT, 2010/Australia | 3 | 0 | 3 | 0 | 0 | 0 | 0 | 0 | 2 | 1 | 3 | 0 |
| Lapphra K, 2010/Canada | 1 | 1 | 0 | 0 | 0 | 1 | 0 | 0 | 0 | 0 | 1 | 0 |
| Park YJ, 2010/Korea | 1 | 0 | 1 | 1 | 0 | 0 | 0 | 0 | 0 | 0 | 1 | 0 |
| Arcondo MF, 2011/Argentina | 1 | 0 | 1 | 0 | 0 | 0 | 0 | 0 | 1 | 0 | 1 | 0 |
| Choe YJ, 2011/Korea | 22 | 0 | 22 | 22 | 0 | 0 | 0 | 0 | 0 | 0 | 22 | 0 |
| De Almeida DF, 2011/Brazil | 1 | 0 | 1 | 1 | 0 | 0 | 0 | 0 | 0 | 0 | 1 | 0 |
| Campanharo FF, 2012/Brazil | 1 | 0 | 1 | 1 | 0 | 0 | 0 | 0 | 0 | 0 | 1 | 0 |
| Lee ST, 2011/Korea | 1 | 1 | 0 | 0 | 0 | 1 | 0 | 0 | 0 | 0 | 1 | 0 |
| Fernandes AF, 2011/Brazil | 1 | 1 | 0 | 0 | 0 | 1 | 0 | 0 | 0 | 0 | 1 | 0 |
| Williams SE, 2011/USA | 219 | 219 | 0 | 98 | 23 | 17 | 7 | 7 | 0 | 67 | 219 | 0 |
| Willi B, 2011/Switzerland | 1 | 1 | 0 | 0 | 0 | 0 | 1 | 0 | 0 | 0 | 1 | 0 |
| Kawashima H, 2012/Japan | 33 | 33 | 0 | 0 | 0 | 0 | 33 | 0 | 0 | 0 | 33 | 0 |

CN-GBS: cranial neuropathies-Guillain Barre Syndrome;S-ES: seizures-Epileptic status; ANE: acute necrotizing encephalitis;ADEM: acute diseminated encephalomyelitis

** Neuropsychiatric symptoms, neuromuscular disorder, cerebellitis, retinitis, etc. # number of cases.

**Supplementary Table 2. Description of the different neurological symptoms related to influenza infection**

|  |  | **Group of patients** | | **Infectious neurological events** | | | | | | | **Clinical outcome** | |
| --- | --- | --- | --- | --- | --- | --- | --- | --- | --- | --- | --- | --- |
| **Reference/ Country** | **Number of patients** | **Pediatrics** | **Adults** | **#CN-GBS** | **#S-ES** | **#ADEM** | **#Encefalitis-ANE** | **#Stroke** | **#Myelitis** | **#Others**** | **#Survived** | **#Died** |
| CDC 2009/USA | 4 | 3 | 1 | 0 | 0 | 0 | 4 | 0 | 0 | 0 | 4 | 0 |
| D’ Silva D 2009/Australia | 1 | 1 | 0 | 0 | 0 | 0 | 0 | 0 | 0 | 1 | 1 | 0 |
| Lister P, 2009/UK | 1 | 1 | 0 | 0 | 0 | 0 | 1 | 0 | 0 | 0 | 1 | 0 |
| Akins PT, 2010/USA | 1 | 0 | 1 | 0 | 0 | 0 | 1 | 0 | 0 | 0 | 1 | 0 |
| Apok V, 2010/UK | 1 | 1 | 0 | 0 | 0 | 0 | 0 | 0 | 0 | 1 | 0 | 1 |
| Baltagi SA 2010/USA | 3 | 3 | 0 | 0 | 0 | 4 | 0 | 0 | 0 | 0 | 3 | 0 |
| Buccoliero G, 2010/Italia | 2 | 0 | 2 | 0 | 0 | 0 | 2 | 0 | 0 | 0 | 2 | 0 |
| Bustos B, 2010/Chile | 1 | 1 | 0 | 0 | 0 | 0 | 1 | 0 | 0 | 0 | 0 | 1 |
| Blum A, 2010/Israel | 1 | 0 | 1 | 0 | 0 | 0 | 0 | 0 | 0 | 0 | 1 | 0 |
| Calitri C, 2010/Italia | 4 | 4 | 0 | 0 | 1 | 2 | 1 | 0 | 0 | 0 | 4 | 0 |
| Citerio G, 2010/Italy | 1 | 1 | 0 | 0 | 0 | 0 | 1 | 0 | 0 | 0 | 1 | 0 |
| Costiniuk , 2010/Canada | 1 | 1 | 0 | 1 | 0 | 0 | 0 | 0 | 0 | 0 | 1 | 0 |
| Chaari A, 2010/Tunisia | 1 | 0 | 1 | 0 | 0 | 0 | 0 | 0 | 0 | 0 | ND | ND |
| Chen YC, 2010/Taiwan | 1 | 0 | 1 | 0 | 0 | 0 | 1 | 0 | 0 | 0 | 1 | 0 |
| Choi SY, 2010/Korea | 1 | 0 | 1 | 0 | 0 | 0 | 1 | 0 | 0 | 0 | 1 | 0 |
| Davis LE, 2010/USA | 32 | 32 | 0 | 0 | 15 | 0 | 0 | 0 | 0 | 17 | 32 | 0 |
| Ekstrand JJ, 2010/USA | 18 | 18 | 0 | 0 | 0 | 0 | 18 | 0 | 0 | 0 | 1 | 17 |
| Fugate J, 2010/USA | 1 | 0 | 1 | 0 | 0 | 0 | 0 | 1 | 0 | 0 | 1 | 0 |
| González-Duarte A, 2010/México | 1 | 0 | 1 | 0 | 0 | 0 | 1 | 0 | 0 | 0 | 1 | 0 |
| German-Díaz M, 2010/Spain | 1 | 1 | 0 | 0 | 0 | 0 | 0 | 0 | 0 | 1 | 1 | 0 |
| Haktanir A, 2010/Turkey | 1 | 1 | 0 | 0 | 0 | 0 | 1 | 0 | 0 | 0 | 1 | 0 |
| Kitcharoen S, 2010/Thailand | 1 | 0 | 1 | 1 | 0 | 0 | 0 | 0 | 0 | 0 | 1 | 0 |
| Kulkarni R, 2010/India | 1 | 1 | 0 | 0 | 0 | 1 | 0 | 0 | 0 | 0 | 1 | 0 |
| Kutlesa M, 2010/Croatia | 1 | 0 | 1 | 1 | 0 | 0 | 0 | 0 | 0 | 00 | 1 | 0 |
| Li D, 2010/China | 1 |  |  |  |  |  |  |  |  |  |  |  |
| Li X, 2010/China | 1 | 1 | 0 | 0 | 0 | 0 | 1 | 0 | 0 | 0 | 1 | 0 |
| Lyon JB, 2010/USA | 1 | 1 | 0 | 0 | 0 | 1 | 0 | 0 | 0 | 0 | 0 | 1 |
| Iwata, 2010/Japan | 1 | 1 | 0 | 0 | 0 | 0 | 0 | 1 | 0 | 0 | 1 | 0 |
| Mariotti P, 2010/Italy | 1 | 1 | 0 | 0 | 0 | 0 | 1 | 0 | 0 | 0 | 1 | 0 |
| Martin A, 2010/USA | 1 | 1 | 0 | 0 | 0 | 0 | 1 | 0 | 0 | 0 | 0 | 1 |
| Noriega LM, 2010/Chile | 6 | 2 | 4 | 0 | 0 | 4 | 0 | 0 | 0 | 0 | 4 | 0 |
| O’Leary MF, 2010/USA | 1 | 1 | 0 | 0 | 1 | 0 | 0 | 0 | 0 | 0 | 1 | 0 |
| Ormiti F, 2010/Italy | 1 | 1 | 0 | 0 | 0 | 0 | 1 | 0 | 0 | 0 | ND | ND |
| Samuel N, 2010/Israel | 1 | 1 | 0 | 0 | 0 | 0 | 1 | 0 | 0 | 0 | 1 | 0 |
| Sanchez-Torrent, 2010/Spain | 1 | 1 | 0 | 0 | 0 | 1 | 0 | 0 | 0 | 0 | 1 | 0 |
| Sugaya N, 2010/Japan | 257 | 257 | 0 | 0 | 138 | 0 | 112 | 0 | 0 | 3 | 257 | 0 |
| Tan K, 2010/Singapore | 9 | 1 | 8 | 0 | 6 | 0 | 0 | 1 | 0 | 2 | 9 | 0 |
| Webster RI, 2010/Australia | 2 | 2 | 0 | 0 | 0 | 0 | 2 | 0 | 0 | 0 | 2 | 0 |
| Yang J, 2010 | 1 | 0 | 1 | 0 | 0 | 1 | 0 | 0 | 0 | 0 | 1 | 0 |
| Al-Baghli F, 2011/Kuwait | 1 | 1 | 0 | 0 | 0 | 0 | 1 | 0 | 0 | 0 | 1 | 0 |
| Augarten A, 2011/Israel | 1 | 1 | 0 | 0 | 0 | 0 | 0 | 0 | 0 | 1 | 1 | 0 |
| Asadi-Pooya A, 2011/Iran | 23 | 2 | 21 | 11 | 1 | 0 | 5 | 0 | 0 | 6 | 18 | 5 |
| Blumental S, 2011/Belgium | 22 | 22 | 0 | 0 | 22 | 0 | 0 | 0 | 0 | 0 | ND | ND |
| Cheng A, 2011/Taiwan | 1 | 0 | 1 | 0 | 0 | 0 | 1 | 0 | 0 | 0 | 1 | 0 |
| Cheng X, 2011/China | 2 | 2 | 0 | 0 | 2 | 0 | 0 | 0 | 0 | 0 | 0 | 2 |
| del Rosal T, 2011/Spain | 12 | 12 | 0 | 0 | 7 | 0 | 5 | 0 | 0 | 0 | 12 | 0 |
| Farooq , 2011/USA | 6 | 6 | 0 | 0 | 2 | 0 | 3 | 0 | 0 | 1 | 4 | 2 |
| Fearnley RA, 2011/UK | 1 | 0 | 1 | 0 | 0 | 0 | 0 | 1 | 0 | 0 | 1 | 0 |
| Frobert E, 2011/Francia | 14 | 14 | 0 | 8 | 0 | 0 | 4 | 1 | 0 | 1 | 13 | 1 |
| Hasegawa, 2011/Japan | 16 | 15 | 1 | 0 | 0 | 0 | 16 | 0 | 0 | 0 | 13 | 3 |
| Kahle K, 2011/USA | 1 | 0 | 1 | 0 | 0 | 0 | 1 | 0 | 0 | 0 | 0 | 1 |
| Kedia S, 2011/USA | 23 | 23 | 0 | 0 | 0 | 2 | 1 | 0 | 0 | 20 | 19 | 4 |
| Kormur M, 2011/Turkey | 1 | 1 | 0 | 0 | 0 | 0 | 1 | 0 | 0 | 0 | 1 | 0 |
| Kumakura, 2011/Japan | 1 | 1 | 0 | 0 | 0 | 0 | 1 | 0 | 0 | 0 | 1 | 0 |
| Launay E, 2011/Canada | 19 | 19 | 0 | 0 | 0 | 0 | 19 | 0 | 0 | 0 | 19 | 0 |
| Lonchindarat S, 2011/Thailand | 3 | 3 | 0 | 0 | 0 | 0 | 3 | 0 | 0 | 0 | 3 | 0 |
| Linden K, 2011/Germany | 1 | 1 | 0 | 0 | 0 | 0 | 0 | 1 | 0 | 0 | 1 | 0 |
| Lung DC, 2011/China | 1 | 1 | 0 | 0 | 0 | 0 | 1 | 0 | 0 | 0 | 0 | 1 |
| McMullan B, 2011/New Zealand | 3 | 3 | 0 | 0 | 1 | 0 | 1 | 0 | 1 | 0 | 3 | 0 |
| Mukherjee A, 2011/USA | 5 | 2 | 3 | 0 | 2 | 0 | 0 | 1 | 2 | 0 | 0 | 5 |
| Nowak DA, 2011/Germany | 2 | 0 | 2 | 0 | 0 | 0 | 0 | 0 | 2 | 0 | 2 | 0 |
| Omari I, 2011/Israel | 6 | 6 | 0 | 0 | 0 | 0 | 5 | 0 | 0 | 1 | 6 | 0 |
| Özdemir H, 2011/Turkey | 1 | 1 | 0 | 0 | 0 | 0 | 0 | 0 | 0 | 1 | 1 | 0 |
| Ozkan M, 2011/Turkey | 17 | 17 | 0 | 1 | 13 | 0 | 3 | 0 | 0 | 0 | 17 | 0 |
| Poeppl W, 2011/Austria | 19 | 8 | 11 | 3 | 0 | 0 | 0 | 0 | 0 | 16 | ND | ND |
| Rellosa N, 2011/USA | 3 | 3 | 0 | 1 | 0 | 0 | 2 | 0 | 0 | 0 | 3 | 0 |
| Sachedina S, 2011/UK | 26 | 26 | 0 | 0 | 5 | 0 | 17 | 0 | 0 | 4 | ND | ND |
| Sandoval Gutierrez JL, 2011/México | 1 | 0 | 1 | 0 | 0 | 0 | 0 | 1 | 0 | 0 | 1 | 0 |
| Spalice A, 2011/Italia | 1 | 1 | 0 | 0 | 0 | 0 | 1 | 0 | 0 | 0 | 0 | 1 |
| Surana P, 2011 | 5 | 5 | 0 | 0 | 1 | 1 | 3 | 0 | 0 | 0 | 5 | 0 |
| Thampi N, 2011/Canada | 1 | 1 | 0 | 0 | 0 | 0 | 1 | 0 | 0 | 0 | 0 | 1 |
| Tsai CK, 2011 | 1 | 0 | 1 | 0 | 0 | 0 | 1 | 0 | 0 | 0 | 0 | 1 |
| Vilà de Muga M, 2011/Spain | 39 | 39 | 0 | 0 | 0 | 0 | 0 | 0 | 0 | 39 | 39 | 0 |
| Wang A, 2011/China | 1 | 0 | 1 | 0 | 0 | 1 | 0 | 0 | 0 | 0 | 1 | 0 |
| Yildizdaş D, 2011/Turkey | 8 | 8 | 9 | 0 | 2 | 1 | 4 | 0 | 0 | 1 | 7 | 1 |
| Athauda D, 2012/UK | 1 | 1 | 0 | 0 | 0 | 0 | 1 | 0 | 0 | 0 | 1 | 0 |
| Cortese A, 2012/Italia | 1 | 0 | 1 | 1 | 0 | 0 | 0 | 0 | 0 | 0 | ND | ND |
| Honorat R 2012/France | 1 | 0 | 1 | 0 | 0 | 1 | 0 | 0 | 0 | 0 | 1 | 0 |
| Locuratolo N, 2012/Italia | 1 | 0 | 1 | 0 | 0 | 0 | 1 | 0 | 0 | 0 | 1 | 0 |
| Kwon S, 2012/Korea | 25 | 25 | 0 | 0 | 20 | 0 | 4 | 0 | 0 | 1 | 24 | 1 |
| Okumura A, 2012/Japan | 10 | 10 | 0 | 0 | 4 | 0 | 4 | 0 | 0 | 2 | 10 | 0 |
| Elliot E, 2012/Australia | 35 | 35 | 0 | 1 | 21 | 0 | 11 | 1 | 1 | 0 | 33 | 2 |
| Glaser C, 2012/USA | 427 | 423 | 4 | 1 | 45 | 0 | 29 | 0 | 0 | 352 | 427 | 0 |
| Yeo L, 2012/Singapore | 1 | 0 | 1 | 0 | 1 | 0 | 0 | 0 | 0 | 0 | 1 | 0 |
| Vasconcelos A, 2012/Portugal | 1 | 1 | 0 | 1 | 0 | 0 | 0 | 0 | 0 | 0 | 1 | 0 |
| Burad J 2012/Oman | 1 | 0 | 1 | 0 | 0 | 0 | 0 | 1 | 0 | 0 | 1 | 0 |
| Tokuhira N, 2012/Japan | 26 | 26 | 0 | 0 | 13 | 0 | 13 | 0 | 0 | 0 | 26 | 0 |
| Khandaker G, 2012/Australia | 49 | 49 | 0 | 0 | 39 |  | 9 | 1 | 0 | 0 | 47 | 2 |
| Rifkin L, 2012/USA | 1 | 0 | 1 | 0 | 0 | 0 | 0 | 0 | 0 | 1 | 1 | 0 |
| Kawashima H 2012/Japan | 120 | 120 | 0 | 0 | 0 | 0 | 120 | 0 | 0 | 0 | 104 | 16 |
| Alsanosi A, 2012/Saudi Arabia | 2 | 2 | 0 | 0 | 0 | 0 | 0 | 0 | 0 | 2 | 2 | 0 |
| Pula J, 2012/USA | 1 | 0 | 1 | 0 | 0 | 0 | 1 | 0 | 0 | 0 | 1 | 0 |

CN-GBS: cranial neuropathies-Guillain Barre Syndrome;S-ES: seizures-Epileptic status; ANE: acute necrotizing encephalitis;ADEM: acute diseminated encephalomyelitis, ND. No data

** Neuropsychiatric symptoms, neuromuscular disorder, cerebellitis, retinitis, etc. # number of cases
